# Clinical performance of the ethanol-wet bonding technique with different adhesive systems in noncarious cervical lesion restorations: a 6-year randomized clinical trial

**DOI:** 10.1007/s00784-026-06930-8

**Published:** 2026-06-03

**Authors:** Luana dos Santos Souza, Maria Fernanda Monnerat, Maurício Yugo Souza, Marcella Batista Rocha, Taciana Marco Ferraz Caneppele, Eduardo Bresciani

**Affiliations:** 1 Institute of Science and Technology, Department of Restorative Dentistry, São Paulo State University - UNESP, Avenida Engenheiro Francisco José Longo, 777, Jardim São Dimas, São José dos Campos, 12245-000 SP Brasil; 2 Centre for Rural Denstirstry and Oral Health, School of Dentistry and Medical Sciences, Charles Sturt University, Orange, NSW 2800 Australia

**Keywords:** Dentin, Ethanol, Adhesives, Controlled clinical trial

## Abstract

**Objectives:**

This randomized clinical trial evaluated the six-year clinical performance of the ethanol-wet bonding technique (EWBT) combined with different adhesive protocols in non-carious cervical lesions (NCCLs). The research question was whether EWBT improves long-term restoration retention compared with conventional water-wet bonding.

**Material and methods:**

Patients presenting at least one NCCL, regardless of lesion etiology (abrasion, erosion, abfraction, or mixed), were included. A total of 148 NCCLs were randomized at the restoration level into four groups: [NE] water-wet bonding with a three-step etch-and-rinse adhesive (Adper Scotchbond Multi-Purpose, 3M ESPE); [EMP] EWBT + Adper Scotchbond Multi-Purpose (3M ESPE); [EB] EWBT + hydrophobic bonding resin (Adper Scotchbond Multi-Purpose Bond, 3M ESPE); and [EU] EWBT + universal adhesive (Single Bond Universal, 3M ESPE). EWBT consisted of applying 100% ethanol actively for 60 s to dentin prior to adhesive application. All restorations were placed using a nanohybrid composite resin (Filtek Z350 XT, 3M ESPE). Clinical evaluations were performed at baseline, 6, 18 months and after six years using modified USPHS criteria. The unit of analysis was the restoration. Intergroup comparisons at each time point were performed using Fisher’s exact test, and survival analysis was conducted using Kaplan–Meier estimates and log-rank tests (α = 0.05).

**Results:**

After six years, 42 patients (108 restorations) were clinically evaluated (72.9% recall), while all 148 restorations were included in the intention-to-treat analysis. Survival rates were 88.9% (NE), 81.1% (EU), 77.8% (EMP), and 48.7% (EB). The EB group showed significantly lower survival than all other groups (log-rank, *p* = 0.008). No significant differences were observed among the hydrophilic protocols (*p* > 0.05).

**Conclusions:**

The effectiveness of EWBT is influenced by the hydrophilicity of the adhesive system. Hydrophilic adhesives showed stable long-term performance regardless of ethanol pretreatment, whereas the use of EWBT with a purely hydrophobic bonding resin resulted in significantly reduced retention.

**Clinical relevance:**

EWBT does not improve longevity when used with hydrophilic adhesives but may compromise performance when associated with simplified hydrophobic bonding strategies.

**Supplementary Information:**

The online version contains supplementary material available at 10.1007/s00784-026-06930-8.

## Introduction

The hybrid layer is formed by the infiltration of adhesive monomers into the demineralized dentin matrix, and the quality of this interaction determines the long-term stability of the resin–dentin bond [1]. Despite advances in adhesive formulations, the hybrid layer remains the weakest component of the adhesive interface. Its gradual degradation over time compromises the marginal integrity of restorations, leading to microleakage, secondary caries, and eventual clinical failure [2–5]. Therefore, improving the structure and durability of this layer remains a central goal in adhesive dentistry.

The long-term stability of the hybrid layer is influenced by the chemical composition and structural complexity of adhesive systems. Simplified adhesives, such as one-step self-etch systems and two-step etch-and-rinse systems in which hydrophilic and hydrophobic components are combined in a single bottle, have been associated with greater susceptibility to hydrolytic degradation. The presence of hydrophilic monomers and residual solvents increases water sorption and permeability of the adhesive layer, facilitating fluid diffusion across the interface and accelerating degradation over time [6].

During the bonding procedure, resin monomers infiltrate the demineralized collagen matrix to form the hybrid layer. However, incomplete infiltration or the presence of residual water may leave exposed collagen fibrils susceptible to hydrolytic and enzymatic degradation, particularly due to the activation of endogenous matrix metalloproteinases (MMPs). In addition, the physicochemical characteristics of adhesive monomers and solvents play an important role in hybrid layer formation and bond durability. Adhesives containing more hydrophobic monomers generally require a relatively dry substrate to form a stable hybrid layer, whereas hydrophilic systems are less dependent on substrate dryness but are more prone to water sorption and hydrolysis. Proper evaporation of solvents such as ethanol, acetone, or water is therefore essential for adequate polymer network formation and long-term stability of the adhesive interface [7, 8].

The ethanol-wet bonding technique (EWBT) has been introduced as a promising approach to improve the infiltration of hydrophobic resin monomers into the demineralized collagen network, aiming to increase the stability of the hybrid layer and prolong bond durability [9, 10]. Ethanol acts as a chemical dehydrating agent, replacing residual water within the collagen fibrils and promoting the formation of a more hydrophobic environment. Because ethanol exhibits a higher solvent capacity and lower hydrogen-bonding potential than water, it enhances resin monomer diffusion and reduces the susceptibility of the interface to hydrolysis [11]. Furthermore, ethanol has been shown to exert an inhibitory effect on matrix metalloproteinases (MMPs), enzymes responsible for the degradation of the collagen matrix, without inducing additional pulpal inflammation compared with conventional water-wet bonding [12, 13]. By promoting a more hydrophobic environment within the collagen network, EWBT may facilitate the infiltration of hydrophobic resin monomers and potentially improve bonding stability, particularly in challenging substrates such as the sclerotic dentin frequently observed in NCCLs.

Non-carious cervical lesions (NCCLs) are characterized by the loss of dental hard tissue at the cementoenamel junction in the absence of caries. Their etiology is considered multifactorial and involves the interaction of several mechanisms, including abrasion related to toothbrushing habits, erosion caused by chemical dissolution from dietary or intrinsic acids, and abfraction associated with occlusal stress and tooth flexure. In clinical practice, these mechanisms frequently coexist, resulting in lesions produced by mixed etiological factors rather than by a single isolated process [14]. Since these lesions frequently result from the interaction of multiple etiological factors, the identification of a single predominant cause is often challenging in clinical settings.

The diagnostic assessment of NCCLs represents a clinical challenge because the morphological characteristics of the lesions do not always allow a clear distinction between the different etiological mechanisms. Wedge-shaped defects have traditionally been associated with abfraction, whereas smooth and shallow lesions are more frequently related to erosion or abrasion; however, such patterns are not definitive. Therefore, the diagnostic process should include a comprehensive clinical evaluation, assessment of occlusal conditions, and a detailed investigation of behavioral and dietary habits in order to identify potential contributing factors [15]. In addition to restorative treatment, the management of etiological factors is essential to prevent lesion progression and restoration failure. Clinical strategies may include occlusal adjustment when excessive stress is identified, dietary counseling to reduce acidic exposure, and modifications in oral hygiene techniques, particularly the use of less abrasive toothbrushing practices.

Although erosive challenges are recognized as an important etiological factor in the development of non-carious cervical lesions (NCCLs), their clinical presentation is heterogeneous, ranging from localized to more generalized patterns of tooth wear. In cases where erosion is ongoing, the dentin substrate may undergo continuous chemical modification, resulting in altered structural and physicochemical properties that can potentially affect adhesive performance. Therefore, caution is warranted when interpreting bonding outcomes in NCCLs associated with active erosive processes, as the characteristics of the underlying dentin may differ substantially from those observed in non-eroded substrates [16]. In these cavities, adhesion relies almost exclusively on micromechanical interlocking between the adhesive system and the dentin substrate.

The dentin substrate in NCCLs frequently exhibits structural alterations such as dentin sclerosis and surface hypermineralization. These changes result from the gradual occlusion of dentinal tubules by mineral deposits and the formation of a highly mineralized superficial layer, which may reduce dentin permeability and compromise the infiltration of adhesive monomers. Accordingly, the bonding effectiveness in NCCLs can be lower and more technique-sensitive compared with sound dentin [17]. Therefore, NCCLs represent a reliable clinical model to assess the actual performance and durability of adhesive procedures under intraoral conditions [18].

Although laboratory studies and short-term clinical trials have demonstrated encouraging results for the ethanol-wet bonding technique [9, 19–21], long-term clinical evidence remains limited. Most available studies report follow-up periods ranging from 6 to 24 months, which are insufficient to capture the gradual degradation processes that occur within the hybrid layer over time. Degradation of the adhesive interface is a slow and cumulative phenomenon influenced by factors such as occlusal stress, water sorption, and enzymatic activity, which may compromise bond durability over extended periods [2, 22]. Therefore, the interaction between EWBT, adhesive composition, and dentin substrate characteristics is critical to understanding its potential clinical effectiveness. The ethanol-wet bonding technique has been proposed to improve bond durability by promoting dehydration of the collagen network and facilitating the infiltration of hydrophobic resin monomers, resulting in a more stable and less permeable hybrid layer [10, 11].

The present study reports the six-year clinical outcomes of a randomized controlled trial evaluating different ethanol-wet bonding protocols compared with conventional water-wet bonding and standard adhesive systems in non-carious cervical lesions. This extended follow-up provides valuable evidence regarding the clinical performance and stability of the ethanol-wet bonding technique over time. The null hypothesis tested was that the use of EWBT would not result in superior bond durability or clinical retention compared with conventional adhesive procedures after six years of service.

## Methodology

This study is part of the same randomized controlled clinical trial previously published by Souza et al. (2019) [19] which reported the six-month outcomes of restorations produced with the ethanol-wet bonding technique (EWBT). The study was conducted between March 2017 and November 2023, with approval from the local Institutional Review Board (protocol number: 2.022.383) and registration in the Registro Brasileiro de Ensaios Clínicos (ReBEC; RBR-5hncr3); all participants provided informed consent prior to enrollment, and the six-year follow-up adhered to the same ethical approval, as well as to CONSORT guidelines and the principles of the Declaration of Helsinki.

The current report presents the results of the six-year follow-up of the same cohort. The study design, inclusion and exclusion criteria, randomization process, restorative protocols, and baseline evaluation procedures were described in detail in the initial report, and the study design, eligibility criteria, randomization process, restorative procedures, and evaluation methods are summarized below to ensure methodological transparency. In the present study, lesions were not classified according to a specific etiological mechanism, as patients commonly presented with combined risk factors. This approach aimed to reproduce the clinical reality in which NCCLs are typically multifactorial and not easily attributable to a single cause.

### Sample size calculation

For this study, the sample size was calculated using an online statistical website - Sealed Envelope Ltda (www.sealedenvelop.com). For power calculation, equivalence trial function under binary outcome was selected, and the following parameters were chosen: α = 5%, power at 80%, success of control and experimental group at 96%, and limit of equivalence at 15% [21]. Sample size was estimated at 30 restorations per group. Considering the potential loss of experimental units during long-term follow-up, an additional margin was incorporated into the recruitment process to ensure that the minimum required number of restorations per group would be maintained throughout the study. Thus, a total of 35 restorations per group were planned, resulting in 140 restorations. During participant recruitment, additional eligible lesions were identified and included, resulting in a final baseline sample of 148 restorations.

### Study design and participants

**Fig. 1:**
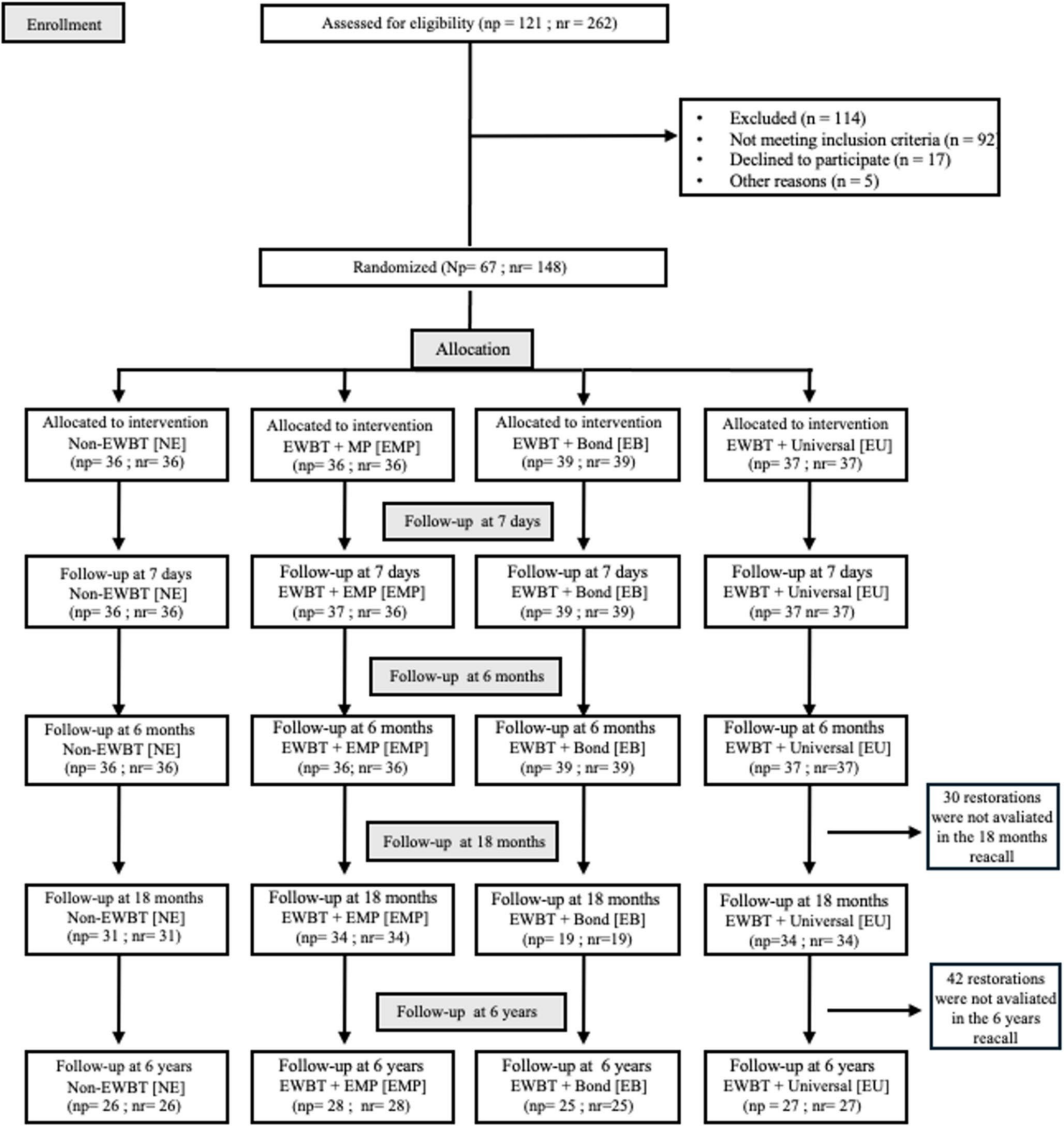
Flowchart. Flow diagram wit details of study phases; EBWT: Ethanol-Wet Bonding Techinique; MP: Adper Scotchbond Multi-Purpose; Bond: Third step of Adper Scotchbond Multi-Purpose; Universal: Single Bond Universal; Np: number of patients included in the study; np: number of patients in each group (one patient is allocated in more than one group); nr: number of restorations

This study was designed as a randomized, double-blind, controlled clinical trial. Adult patients presenting at least one non-carious cervical lesion (NCCL) in permanent canines or premolars were recruited. A total of 67 participants, contributing 148 restorations, were enrolled and allocated to four experimental groups according to the bonding strategy employed: conventional water-wet bonding with a three-step etch-and-rinse adhesive (NE); ethanol-wet bonding combined with a three-step etch-and-rinse adhesive including a hydrophilic primer and hydrophobic bonding resin (EMP); ethanol-wet bonding combined with a hydrophobic bonding resin only (EB); and ethanol-wet bonding combined with a universal adhesive (EU) (Fig. 1).

Eligibility criteria included age ≥ 18 years; presence of one to four non-carious lesions in canines or premolars with a minimum depth and cervico-occlusal height of 1 mm (measured with a graduated periodontal probe); presence of adjacent and antagonistic teeth with occlusal contact; absence of chronic systemic diseases and relevant allergies; and absence of periodontal disease (probing depth ≤ 3 mm and absence of bleeding on probing - periodontal status was assessed clinically by probing depth and bleeding on probing), and a good oral health was defined as absence of untreated caries, absence of active periodontal disease, and adequate oral hygiene. Exclusion criteria were medical conditions or known allergies that could compromise participant safety; use of removable prostheses with clasps on the study teeth; presence of carious lesions or non-vital teeth; and severe parafunctional habits (severe parafunctional habits such as bruxism or clenching in patients who did not use occlusal splints). All participants received verbal and written information regarding the study objectives, procedures, potential risks, and benefits, and provided written informed consent prior to enrollment. Participants were informed of their right to withdraw from the study at any time without consequences.

### Randomization and allocation concealment

Randomization was performed “by chance” using an envelope containing the study protocols prior to the allocation of treatments. Randomization occurred at the restoration level. Eligible participants had a minimum of one and a maximum of four non-carious cervical lesions. To enable robust intra-individual comparisons, a strict “no repetition” allocation rule was followed: while a single patient could receive multiple restorations, each lesion was randomly assigned to a different adhesive protocol (NE, EU, EMP, or EB). Consequently, no participant received the same treatment more than once.

When a participant had more than one eligible lesion, a predefined tooth arch positioning sequence determined the order of allocation. The tooth in the quadrant with the lowest numerical order (e.g., the first quadrant) and positioned most mesially within that quadrant was assigned first. This standardized sequence was applied consistently to all participants with multiple lesions to minimize allocation bias and ensure methodological transparency.

### Restorative procedures

Before the restorative procedures, participants received standardized oral hygiene instructions, including recommendations regarding toothbrushing technique and toothbrush selection. All cavities were submitted to professional pumice prophylaxis, using a polishing brush. For the restorative procedure, isolation was done with a lip retractor, cotton rolls and a gingival displacement cord and a saliva retractor. A bevel was not placed before the restoration. Briefly, after 37% phosphoric acid etching for 15 s, 100% ethanol was actively applied for 60 s in EWBT groups and the excess ethanol was air-dried prior to adhesive application. The adhesive systems were then applied as recommended by the manufacturers, according to each group:

- Control group [NE]: Non-EBWT + three step etch-and-rinse (Scotchbond Multi Purpose, Solventum [MP]) primer was applied to etched substrate and dried for 5 s + bond was actively applied for 20 s, followed by a slight blow of air for 5 s and light polymerizing for 10 s.
- EBWT + MP [EMP]: EWBT + three step etch-and-rinse (Scotchbond Multi Purpose, Solventum [MP]) 100% ethanol was actively applied for 60 s + primer was applied to etched substrate and dried for 5 s + bond was actively applied for 20 s, followed by a slight blow of air for 5 s and light polymerizing for 10 s.
- EBWT + Bond [EB]: EWBT + thrid step of the etch-and-rinse adhesive (Scotchbond Multi Purpose, Solventum [MP]) 100% ethanol was actively applied for 60 s + bond actively applied for 20 s, followed by a slight blow of air for 5 s and light polymerizing for 10 s.
- EBWT + Universal Adhesive [EU]: EWBT + Universal adhesive (Scotchbond Universal, Solventum) 100% ethanol was actively applied for 60 s + universal adhesive was actively applied for 20 s, followed by a slight blow of air for 5 s and light polymerizing for 10 s.

All restorations were performed with a nanohybrid resin composite (Filtek Z350 XT, Solventum) (Table 1) using the incremental technique with Radii-Cal LED curing unit (SDI Limited, Bayswater, VIC, Australia) with 900 mW/cm², for 20 s on each layering and an extra 40 s in the last one. Most of restorations were finished with two increments. Finishing and polishing were performed after seven days using fine diamond burs and Sof-Lex™ discs (Solventum).

**Table 1:** Materials used in the study

Material	Manufacturer	Composition
37% Phosphoric acid (Condac 37%)	FGM, Brazil	37% phosphoric acid aqueous solution
Adper Scotchbond Multi-Purpose	Solventum (3 M/ESPE), Sumaré, Brazil	Conventional adhesive system (3-steps): HEMA, water, ethanol, Bis-GMA, HEMA, photoinitiators
Single Bond Universal	Solventum (3 M/ESPE), Sumaré, Brazil	Universal adhesive (one-step): Bis-GMA, HEMA, 10-MDP, silane, water, ethanol, photoinitiators
Filtek Z350 XT	Solventum (3 M/ESPE), Sumaré, Brazil	Nanoparticulate composite resin, Bis-GMA, UDMA, Bis-EMA, TEGDMA, sílica and zircônia (± 72 wt% nanoparticles and nanoclusters)

Materials used in the study and their manufacturers and main chemical compositions. *HEMA,* 2-hydroxyethyl methacrylate; *Bis-GMA,* bisphenol A-glycidyl methacrylate; *UDMA,* urethane dimethacrylate; *Bis-EMA,* ethoxylated bisphenol A dimethacrylate; *TEGDMA,* triethylene glycol dimethacrylate; *10-MDP,* 10-methacryloyloxydecyl dihydrogen phosphate

### Calibration and blinding

To ensure consistent and reliable clinical assessments according to the modified USPHS criteria, two independent and experienced dentists underwent a rigorous calibration process. During the training phase, the evaluators reviewed representative photographs illustrating each score for all evaluated criteria. Furthermore, they independently assessed 10 patients who were not enrolled in the actual clinical trial, over the course of two consecutive days. The clinical evaluations of the study participants were initiated only after an intra-examiner and inter-examiner agreement level of at least 85% was achieved [23].

Regarding blinding, the operator responsible for the restorative procedures could not be blinded due to the distinct clinical application steps required for each adhesive protocol and the ethanol-wet bonding technique. Nevertheless, both the independent evaluators and the patients remained strictly blinded to the allocation groups during the baseline and throughout all subsequent recall evaluations.

### Evaluation procedures

Restorations were re-evaluated after six years using the same criteria adopted at baseline, six-month and 18 months recalls. Two calibrated examiners, blinded to the group allocation, performed all evaluations according to the USPHS modified criteria (Table 2), assessing retention, marginal adaptation, marginal discoloration, secondary caries, anatomic form, surface texture, and postoperative sensitivity. Discrepancies between evaluators were resolved by consensus. The evaluation of the restorations was performed at baseline (one week), and after 6, 18 months and 6 years (72–83 months).

The six-year follow-up, however, was not restricted to a single fixed time point at exactly 72 months. Instead, due to unavoidable constraints related to the COVID-19 pandemic and subsequent interruptions in clinical access, this recall was carried out over an extended interval, ranging from 72 to 83 months. Thus, each patient’s event time was recorded at the actual observation month, preserving the temporal accuracy of the data rather than artificially clustering outcomes at a fixed time point. The categorization of this interval as a “6-year follow-up” was adopted for clinical interpretability and consistency with prior reports, while the survival curve reflects the real distribution of observation times across this period. The same calibration process used at baseline (Kappa = 0.61) was repeated prior to the six-year assessment.

**Table 2:** USPHS modified criteria

USPHS modified criteria	
Retention	
Alfa (A)	No restorative material loss
Charlie (C)	Partial or complete loss of restorative material
Marginal discoloration	
Alfa (A)	No discoloration between tooth structure and restorative material
Bravo (B)	Marginal discoloration which can be polished away
Charlie (C)	Discoloration in interface restorative material and tooth, not able to polish
Marginal adaptation	
Alfa (A)	Closely adapted, no detectable margin
Bravo (B)	Detectable marginal discrepancy clinically acceptable
Charlie (C)	Marginal crevice, clinically unacceptable
Delta (D)	Mobile restoration, partially ou totally fractured
Secondary caries	
Alfa (A)	No caries present
Charlie (C)	Caries present
Anatomic form	
Alfa (A)	Continuous, well contoured
Bravo (B)	Slight discontinuity or under contoured without dentin exposure, clinically acceptable
Charlie (C)	Discontinuous, severe under contoured, with dentin exposure, clinically unacceptable
Post-operative sensitivity	
Alfa (A)	No post-operative sensitivity
Bravo (B)	Sensitive but with intensity decreasing
Charlie (C)	Constant sensitivity, without intensity decreasing
Surface texture	
Alfa (A)	Textures such as enamel
Bravo (B)	Textures such as composite resin
Charlie (C)	Surface with pares or cracks, with dental pick retention

### Statistical analysis

Randomization was performed at the restoration level, allowing a single patient to receive more than one type of adhesive protocol. Consequently, the statistical analysis treated each restoration as an independent unit. It should be noted that the non-parametric tests employed did not statistically account for the potential cluster effect (multiple restorations within the same individual), which is acknowledged as a limitation of the present study. The statistical analysis followed the intention-to-treat (ITT) protocol, in accordance with CONSORT guidelines [24], which involved all restorations initially randomized, including those from patients who were not evaluated at the specified follow-up periods. To address these dropouts, missing data were handled using the Last Observation Carried Forward (LOCF) imputation method, wherein the clinical score of the last valid evaluation was repeated for the subsequent missing periods.

Because the modified USPHS criteria generate categorical and ordinal data, and a normal distribution could not be assumed, non-parametric tests were applied. For the primary outcome of restoration retention, cumulative survival rates were estimated using Kaplan-Meier analysis. Cases of patient dropouts were right-censored at the time of their last clinical evaluation, and differences between the survival curves of the four groups were compared using the log-rank test. Statistical analysis of the secondary clinical parameters (marginal discoloration, marginal adaptation, surface texture, and postoperative sensitivity) was performed considering each period of evaluation. Differences in the ratings of the four groups at each specific assessment period (baseline, 6 months, 18 months, and 6 years) were compared using Fisher’s Exact test, followed by pairwise Fisher’s tests for post-hoc comparisons. Additionally, intra-group differences among the evaluation periods within each specific protocol were tested by Friedman’s repeated measures analysis of variance by ranks, followed by the Wilcoxon signed-rank test with Bonferroni adjustment for pairwise comparisons.

In all statistical tests, the significance level was preset at 5% (α = 0.05). All analyses were performed using the R statistical language (RStudio, version 3.4.4, RStudio Team, Boston, MA, USA).

## Results

### Characteristics of included patients

Figure 1 illustrates the CONSORT flowchart detailing the flow of participants throughout the clinical trial. Initially, 121 subjects (comprising 262 non-carious cervical lesions) were assessed for eligibility. From this total, 114 lesions were excluded, primarily for not meeting the inclusion criteria or due to patients declining to participate. Consequently, 67 participants with a total of 148 restorations were enrolled and randomized into the four experimental groups: NE (*n* = 36), EU (*n* = 37), EMP (*n* = 36), and EB (*n* = 39). The baseline demographic data and the clinical characteristics of the restored cavities (tooth type, lesion shape, cavity depth, occlusion, and preoperative sensitivity) were homogeneously distributed among the experimental groups and are detailed in Table 3.

**Table 3:** Baseline demographic and clinical characteristics of the randomized restorations according to the experimental groups

	NE	EU	EMP	EB
	(*n* = 36)	(*n* = 37)	(*n* = 36)	(*n* = 39)
Sex, n (%)				
Male	10 (28%)	11 (30%)	11 (31%)	12 (31%)
Female	26 (72%)	26 (70%)	25 (69%)	27 (69%)
Age (years), n (%)				
18-30	4 (11%)	3 (8%)	5 (14%)	3 (8%)
31-40	9 (25%)	10 (27%)	9 (25%)	11 (28%)
41-50	14 (39%)	15 (41%)	13 (36%)	15 (38%)
51-60	9 (25%)	9 (24%)	9 (25%)	10 (26%)
Tooth type, n (%)				
Maxillary incisors/canines	6 (17%)	7 (19%)	5 (14%)	7 (18%)
Maxillary premolars	11 (31%)	12 (32%)	12 (33%)	12 (31%)
Mandibular incisors/canines	4 (11%)	3 (8%)	5 (14%)	5 (13%)
Mandibular premolars	15 (42%)	15 (41%)	14 (39%)	15 (38%)
Lesion shape				
Wedge	23 (64%)	24 (65%)	22 (61%)	25 (64%)
Saucer	13 (36%)	13 (35%)	14 (39%)	14 (36%)
Cavity extension (depth), n (%)				
< 1.5 mm	21 (58%)	22 (59%)	20 (56%)	23 (59%)
1.5 – 2.5 mm	15 (42%)	15 (41%)	16 (44%)	16 (41%)
Occlusion, n (%)				
Working side	28 (78%)	29 (78%)	27 (75%)	30 (77%)
Non-working side	8 (22%)	8 (22%)	9 (25%)	9 (23%)
Preoperative sensitivity, n (%)				
Yes	11 (31%)	12 (32%)	10 (28%)	13 (33%)
No	25 (69%)	25 (68%)	26 (72%)	26 (67%)

Due to the intra-individual study design (where patients could receive more than one restorative protocol), demographic variables (sex and age) reflect the characteristics of the host patients relative to the number of randomized restorations (n) in each group, not the absolute number of unique individuals. Total randomized restorations = 148. Groups: [NE] - Non-Ethanol, [EMP] - EWBT + Adper Scotchbond Multi-Purpose, [EB] - EWBT + Bond, [EU] - EWBT + Sigle-Bond Universal

The descriptive distribution of the modified USPHS scores for the primary outcome across the different time points (baseline, 6 months [19], 18 months, and 6 years) is presented in Table 4. Additionally, the statistical comparisons of the all evaluation criteria, following the Intention-to-Treat (ITT) principle, are detailed in Supplementary Table.

**Table 4:** Success and failure assessment at baseline and long-term follow-ups under ITT

Group	Outcome	Baseline (7 days)	6 months	18 months	6 years
NE (*n* = 36)	Success	36 (100%)	36 (100%)	36 (100%)	32 (88.9%)
	Failure	0 (0%)	0 (0%)	0 (0%)	4 (11.1%)
EU (*n* = 37)	Success	37 (100%)	36 (97.3%)	35 (94.6%)	30 (81.1%)
	Failure	0 (0%)	1 (2.7%)	2 (5.4%)	7 (18.9%)
EMP (*n* = 36)	Success	36 (100%)	35 (97.2%)	34 (94.4%)	28 (77.8%)
	Failure	0 (0%)	1 (2.8%)	2 (5.6%)	8 (22.2%)
EB (*n* = 38)	Success	37 (97.4%)	29 (76.3%)	23 (60.5%)	18 (47.4%)
	Failure	1 (2.6%)	9 (23.7%)	15 (39.5%)	20 (52.6%)
	*p*-value*	> 0.05	< 0.01	< 0.01	< 0.01

Groups: [NE] - Non-Ethanol, [EMP] - EWBT + Adper Scotchbond Multi-Purpose, [EB] - EWBT + Bond, [EU] - EWBT + Sigle-Bond Universal. *P*-values derived from Fisher's Exact Test comparing the four groups at each specific time point

Over the 6-year follow-up period, patient attrition occurred. At the final evaluation, 42 patients attended the recall, allowing the direct clinical assessment of 108 restorations. This provided an overall recall rate of 72.9%. The patients lost to follow-up could not be evaluated due to changes in contact information, moving to different cities, or failure to attend the scheduled appointments despite multiple contact attempts by the research team. Short-term data regarding the initial restorative procedures and early recalls have been previously published. The present report focuses strictly on the 6-year longitudinal outcomes.

### Retention and survival analysis

The overall survival rate based on restoration retention at the 6-year recall was 73.6% (109 out of 148 restorations). A total of thirty-nine restorations were lost after 6 years of clinical evaluation (4 for NE; 7 for EU; 8 for EMP; and 20 for EB). The 6-year retention rates were 88.9% for NE, 81.1% for EU, 77.8% for EMP, and 48.7% for EB. While all groups exhibited a significant intra-group decrease in retention over the 6-year follow-up (Friedman, *p* < 0.01), the EB protocol demonstrated an early and progressive decline. Inter-group comparisons revealed that the EB group had a significantly higher failure rate compared to the other groups starting at the 6-month evaluation (*p* < 0.01), a trend that persisted through the 18-month and 6-year recalls.

**Fig. 2:**
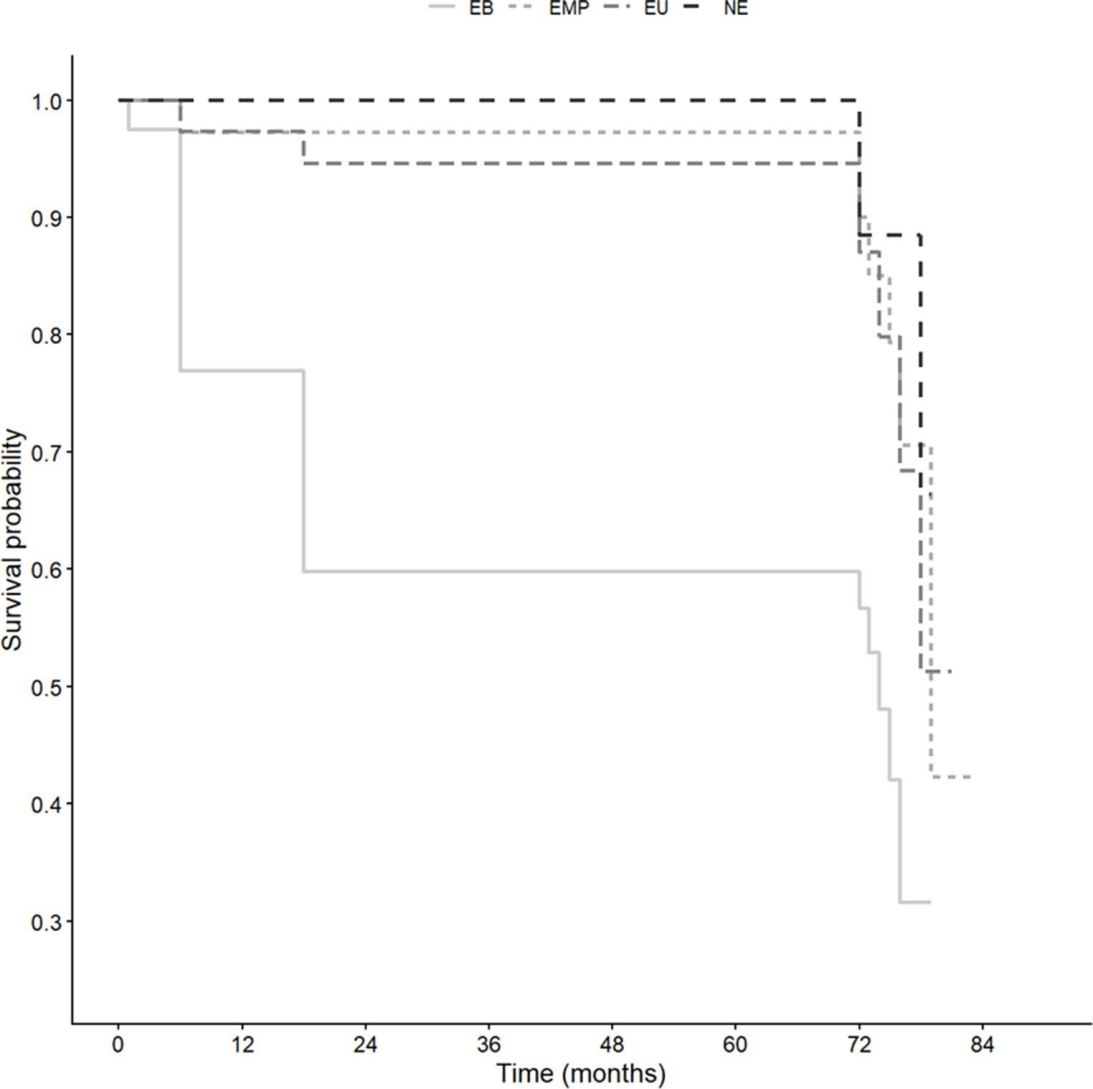
Kaplan–Meier survival curves for the four adhesive protocols (NE, EMP, EB, EU). The final evaluation occurred within a 72-83-month window; extensions beyond 72 months represent the timing of clinical events and censoring across groups. Groups: [NE] - Non-Ethanol, [EMP] - EWBT + Adper Scotchbond Multi-Purpose, [EB] - EWBT + Bond, [EU] - EWBT + Sigle-Bond Universal

Cumulative survival analysis using Kaplan-Meier curves over the 6-year period showed significant overall differences among the adhesive protocols (Log-rank test, *p* = 0.008) (Fig. 2). Pairwise log-rank comparisons confirmed that the EB group presented a significantly lower survival probability than the NE (*p* = 0.030), EU (*p* = 0.005), and EMP (*p* = 0.015) groups. The Kaplan-Meier curves did not show any significant differences among the hydrophilic protocols (NE, EU, and EMP) throughout the entire evaluation period.

### Marginal discoloration

Regarding marginal discoloration, an intra-group significant increase in defects was observed for all adhesive protocols over the 6-year period (Friedman, *p* < 0.01). Inter-group comparisons demonstrated that the EB group presented a significantly higher percentage of marginal discoloration at 6 months (*p* = 0.049) and 18 months (*p* < 0.01) compared to the NE and EU groups. However, at the 6-year follow-up, the success rates dropped across all groups, NE (58.3%), EU (54.1%), EMP (41.7%), and EB (35.9%), with no statistically significant differences among them (Fisher’s exact test, *p* = 0.179).

### Marginal adaptation

A similar pattern was observed for marginal adaptation. Significant intra-group deterioration occurred across all groups over the evaluation period (*p* < 0.01). The EB group showed early and significant degradation, differing from the other protocols at 6 months (*p* = 0.022) and 18 months (*p* < 0.01). After 6 years, the success rates for marginal adaptation were 50.0% for NE, 37.8% for EU, 38.9% for EMP, and 23.1% for EB. Despite the numerical differences, no statistically significant inter-group differences were detected at this final recall (*p* = 0.111).

### Surface texture

For surface texture, a significant degradation was observed within all groups over the 6-year follow-up (*p* < 0.01). The final success rates were 61.1% (NE), 56.8% (EU), 47.2% (EMP), and 33.3% (EB). Pairwise comparisons did not detect statistically significant differences among the adhesive protocols at any of the evaluation periods (*p* > 0.05), indicating a similar pattern of surface wear regardless of the bonding strategy used.

### Post-operative sensitivity

Success for this criterion was defined as the complete absence of post-operative sensitivity. Baseline success rates ranged from 69.2% to 83.3%, with no significant differences among groups (*p* = 0.548). Intra-group variations were observed over time as symptoms fluctuated. Inter-group comparisons revealed significant differences starting at 6 months (*p* = 0.024) and persisting through the 18-month and 6-year evaluations (*p* < 0.01). At the final 6-year recall, the control group (NE) demonstrated significantly higher success rates (83.3%) compared to the EB group (46.2%), while the EU (75.7%) and EMP (77.8%) groups exhibited intermediate clinical performance without differing significantly from the control.

### Other parameters

Throughout the 6-year follow-up period, no surviving restoration showed evidence of secondary caries or alteration of anatomic form. Consequently, all evaluated restorations received an Alpha score for this specific parameters according to the modified USPHS criteria at all recall periods, with no statistically significant differences observed among the experimental groups.

## Discussion

Based on the present findings, the null hypothesis that adhesive pretreatments would not influence the long-term clinical performance was rejected. The hydrophobic protocol associated with ethanol-wet bonding (EB) showed significantly lower survival rates than the other experimental groups (*p* < 0.05). In contrast, the hydrophilic adhesive protocols, whether applied with or without EWBT, showed similar clinical performance to the control group, maintaining high retention and satisfactory marginal adaptation after six years.

The inferior performance observed for the hydrophobic protocol may be explained by the limited infiltration capacity of undiluted hydrophobic bonding resins. These materials present higher viscosity and reduced wettability, which may hinder their penetration into the demineralized collagen network even when ethanol is used as a pretreatment. Consequently, incomplete encapsulation of collagen fibrils may occur, resulting in a structurally weaker hybrid layer and increased susceptibility to degradation over time [22]. While previous short-term clinical data (6 to 18 months) have reported high initial success rates without significant differences among these adhesive strategies, our extended follow-up incorporating intermediate recall data revealed a clear progressive degradation. This limitation was directly reflected in the significantly lower retention rate observed for the EB group, starting as early as 6 months and intensifying up to the six-year evaluation.

Adhesive hydrophilicity plays a central role in hybrid layer formation. Hydrophilic monomers increase the affinity of the adhesive system for moist dentin, facilitating diffusion into the collagen network and improving the initial formation of the hybrid layer. However, this characteristic is also associated with increased water sorption and long-term hydrolytic degradation [22, 25, 26]. In the present trial, hydrophilic adhesives (NE, EU, and EMP) were able to achieve adequate monomer infiltration into the dentin substrate regardless of the use of EWBT. Although the universal adhesive combined with EWBT showed favorable clinical performance, these values were not statistically superior to those observed for the other hydrophilic adhesive protocols. These observations suggest that the long-term clinical behavior of the adhesive interface depends more strongly on the physicochemical characteristics of the adhesive system (such as water affinity) than on the ethanol pretreatment itself [22, 25, 26].

The EWBT was originally proposed for use with hydrophobic adhesives because of its ability to displace water from the collagen matrix and promote monomer infiltration into the dentin network, resulting in a less hydrophilic and more stable hybrid layer [26, 27]. The present 6-year findings, however, clearly indicate that the long-term clinical performance of EWBT depends strongly on the adhesive composition and solvent characteristics.

Clinical trials evaluating the use of EWBT in non-carious cervical lesions have generally reported similar clinical outcomes compared with conventional bonding strategies during short-term follow-up periods. Studies with follow-ups ranging from 6 to 12 months reported no significant differences in retention rates or marginal adaptation among the evaluated adhesive protocols [20, 21]. Similarly, the six-month report of the present clinical trial also demonstrated that the protocol combining EWBT with the hydrophobic bonding resin presented the lowest success rate compared with the other groups [19]. The present six-year results therefore reinforce the tendency observed in the short-term evaluation, suggesting that the use of an undiluted hydrophobic bonding resin may compromise the clinical performance of this technique over time.

Previous studies have suggested that the success of EWBT in conjunction with hydrophobic resins may depend on the use of ethanol-diluted primers, which facilitate the diffusion of hydrophobic monomers into the collagen network [28]. However, such formulations are not commercially available, limiting their applicability in routine clinical practice. Under clinical conditions, the use of commercially available hydrophobic bonding resins without dilution may therefore result in suboptimal infiltration of the demineralized collagen matrix, which could explain the retention observed for the EB group.

Ethanol pretreatment has also been reported to reduce the diameter of collagen fibrils and decrease the overall volume of the collagen matrix by removing interfibrillar water, thereby increasing the interfibrillar spaces available for monomer diffusion [25, 27]. Despite this theoretical advantage, no significant differences were observed between EWBT combined with hydrophilic adhesives and the control group after six years. This finding suggests that when adhesive systems already contain hydrophilic monomers capable of interacting with moist dentin, the additional ethanol pretreatment may not substantially influence the long-term clinical outcome.

EWBT was originally proposed to reduce water sorption, decrease enzyme-mediated collagen degradation, and increase collagen stiffness. However, ethanol application does not completely dehydrate the dentin substrate; residual moisture may remain and interfere with adhesive polymerization. The present 6-year findings clearly indicate that when adhesive systems already contain hydrophilic monomers capable of interacting with moist dentin, the additional ethanol pretreatment does not substantially influence the long-term clinical outcome. For universal adhesives containing the functional monomer 10-MDP—which forms stable calcium salts with hydroxyapatite, the chemical stability of the interface is already enhanced, making the additional EWBT step clinically redundant [27, 28].

This residual moisture may partially explain the reduced retention observed when EWBT was combined with the hydrophobic bonding resin in the present study, fact related to the condition that ethanol application may not completely dehydrate the dentin substrate. Although ethanol is capable of replacing loosely bound water from dentin tubules and collagen microfibrils, residual water may still remain within the interfibrillar spaces of the collagen network, potentially interfering with adhesive infiltration and polymerization [29–33]. In addition, residual moisture has been associated with increased nanoleakage and reduced hybrid layer stability over time [34]. This mechanism may partially explain the reduced retention observed when EWBT was combined with the hydrophobic bonding resin in the present study.

Despite the challenging biomechanical environment of NCCLs, which are often exposed to occlusal stress and dentin sclerosis, the hydrophilic protocols evaluated in this study showed high survival rates in the short term, with values close to 100% at 6 months and only slight reductions at 18 months for most groups. Over time, however, a clearer decline in performance became evident, particularly for the etch-and-rinse approach, while the other strategies remained relatively stable up to 18 months. At the 6-year follow-up, survival rates ranged from approximately 78% to 89% among the best-performing groups, whereas the etch-and-rinse strategy showed a more pronounced reduction. Previous long-term clinical studies on NCCLs have reported similar survival ranges as clinically acceptable, especially given the unfavorable biomechanical conditions associated with these lesions [22–24].

The interaction between ethanol pretreatment and the solvent system of universal adhesives may also influence adhesive performance. Ethanol, as an organic solvent, can promote monomer infiltration by replacing water within the collagen matrix and maintaining interfibrillar spaces open for resin diffusion [27, 32]. However, incomplete solvent evaporation or residual moisture may lead to increased porosity within the hybrid layer, which has been associated with long-term degradation [33–36]. Therefore, the clinical performance of these systems likely reflects a balance between improved monomer diffusion and effective solvent evaporation during adhesive application. Therefore, the clinical performance of these systems likely reflects a balance between improved monomer diffusion and adequate solvent evaporation during adhesive applications.

This study presents some limitations that should be considered when interpreting the results. Randomization was performed at the restoration level, allowing some participants to contribute more than one restoration, which may introduce clustering effects and reduce statistical independence among observations. This intra-individual correlation may have influenced the estimates and should be considered when interpreting the findings. Additionally, missing data resulting from patient dropouts over the 6-year follow-up were handled using an intention-to-treat approach with the last observation carried forward (LOCF) method, which assumes that the clinical condition of a restoration remains unchanged after the last recorded evaluation. Although this approach preserves sample size, it may underestimate failure rates over time. Despite these limitations, the long-term follow-up provides clinically relevant information regarding the durability of adhesive protocols in non-carious cervical lesions.

From a clinical standpoint, the use of EWBT has raised concerns regarding potential postoperative sensitivity. However, a randomized clinical trial demonstrated that the application of EWBT did not increase postoperative hypersensitivity in Class II composite restorations compared with conventional bonding techniques [37]. Although the restorative scenario and cavity configuration differed from those of the present study, these findings support the clinical safety of ethanol-based bonding approaches when properly applied.

Taken together, the results of the present six-year clinical evaluation indicate that the ethanol-wet bonding technique does not necessarily provide superior long-term performance compared with conventional bonding approaches when hydrophilic adhesive systems are employed. Although EWBT did not compromise the performance of these systems, it also did not produce statistically significant improvements in restoration survival. Conversely, the association of EWBT with a purely hydrophobic bonding resin resulted in significantly lower retention rates, suggesting that this combination may not be suitable for routine clinical application. Therefore, the potential benefits of EWBT should be carefully weighed against its additional technique sensitivity.

## Conclusion

Within the limitations of this 6-year clinical trial, the ethanol-wet bonding technique did not significantly improve the long-term clinical performance of NCCL restorations when hydrophilic adhesive systems were used. Comparable survival rates were observed among hydrophilic adhesive protocols regardless of ethanol pretreatment. However, the association of ethanol-wet bonding with a purely hydrophobic bonding resin resulted in significantly lower retention rates. These findings suggest that the clinical performance of ethanol-wet bonding may depend on the physicochemical characteristics of the adhesive system.

## Supplementary Information

Below is the link to the electronic supplementary material.

Supplementary Material 1

## Data Availability

The datasets generated and/or analyzed during the current study are available from the corresponding author upon reasonable request.
