# Supplementary material for: Clinical performance of the ethanol-wet bonding technique with different adhesive systems in noncarious cervical lesion restorations: a 6-year randomized clinical trial
Source: Clin Oral Investig. 2026 Jun 3;30(6):268. doi: 10.1007/s00784-026-06930-8 (PMC13233631; doi:10.1007/s00784-026-06930-8)
Supplement: Supplementary file 1 — Supplementary Material 1 [file 784_2026_6930_MOESM1_ESM.docx]

Supplementary table. Values are expressed as number of successful restorations and percentage (n / %). To maintain the Intention-to-Treat (ITT) principle, missing data due to patient dropouts were handled using the Last Observation Carried Forward (LOCF) method.

| *Criterion/Group* | *Baseline*  *(7 days)* | *6 months* | *18 months* | *6 years*  *(72-78 months)* | *p-value*  *(Friedman)* |
| --- | --- | --- | --- | --- | --- |
| *Retention* |  |  |  |  |  |
| *NE* | 36 (100%) Aa | 36 (100%) Aa | 36 (100%) Aa | 32 (88.9%) Ba | < 0.01 |
| *EU* | 37 (100%) Aa | 36 (97.3%) Aab | 35 (94.6%) Aa | 30 )81.1%) Ba | < 0.01 |
| *EMP* | 36 (100%) Aa | 35 (97.2%) Aab | 34 (94.4%) Aa | 28 (77.8%) Bab | < 0.01 |
| *EB* | 38 (97.4%) Aa | 30 (76.9%) Bb | 24 (61.5%) Cb | 19 (48.7%) Cb | < 0.01 |
| *p-value (Fisher)* | > 0.05 | < 0.01 | < 0.01 | < 0.01 |  |
| *Marginal discoloration* |  |  |  |  |  |
| *NE* | 36 (100%) Aa | 33 (91.7%) Aa | 33 (91.7%) Aa | 21 (58.3%) Ba | < 0.01 |
| *EU* | 37 (100%) Aa | 36 (97.3%) Aa | 35 (94.6%) Aa | 20 (54.1%) Ba | < 0.01 |
| *EMP* | 36 (100%) Aa | 32 (88.9%) Aba | 29 (80.6%) Bab | 15 (41.7%) Ca | < 0.01 |
| *EB* | 38 (97.4%) Aa | 30 (76.9%) Ba | 24 (61.5%) Bb | 14 (35.9%) Ca | < 0.01 |
| *p-value (Fisher)* | > 0.05 | 0.049* | < 0.01 | 0.179 |  |
| *Marginal adaptation* |  |  |  |  |  |
| *NE* | 34 (94.4%) Aa | 34 (94.4%) Aa | 34 (94.4%) Aa | 18 (50%) Ba | < 0.01 |
| *EU* | 34 (91.9%) Aa | 33 (89.2%) Aab | 32 (86.1%) Aa | 14 (38.9%) Ba | < 0.01 |
| *EMP* | 35 (97.2%) Aa | 31 (86.1%) Aa | 31 (86.1%) Aa | 14 (38.9%) Ba | < 0.01 |
| *EB* | 36 (92.3%) Aa | 27 (69.2%) Bb | 20 (51.3%) Cb | 9 (23.1%) Da | <0.01 |
| *p-value (Fisher)* | 0.875 | 0.022 | <0.01 | 0.111 |  |
| *Texture* |  |  |  |  |  |
| *NE* | 36 (100%) Aa | 31 (86.1%) ABa | 26 (72.2%) BCa | 22 (61.1%) Ca | < 0. 01 |
| *EU* | 37 (100%) Aa | 33 (89.2%) Aa | 26 (70.3%) Ba | 21 (56.8%) Ba | < 0.01 |
| *EMP* | 36 (100%) Aa | 36 (100%) Aa | 31 (86.1%) ABa | 25 (69.4%) Ba | < 0.01 |
| *EB* | 38 (97.4%) Aa | 27 (69.2%) Ba | 22 (56.4%) Ba | 13 (33.3%) Ca | < 0.01 |
| *p-value (Fisher)* | > 0.05 | 0.121 | 0.468 | 0.078 |  |
| *Sensivity* |  |  |  |  |  |
| *NE* | 27 (75.0%) Aa | 32 (88.9%) ABa | 35 (97.2%) Ba | 30 (83.3%) ABa | < 0.01 |
| *EU* | 29 (78.4%) ABa | 32 (86.5%) ACa | 33 (89.2%) Ca | 28 (75.7%) Bab | 0.01 |
| *EMP* | 30 (83.3%) Aa | 33 (91.7%) ABa | 34 (94.4%) Ba | 28 (77.8%) ABab | 0.005 |
| *EB* | 27 (69.2%) Aa | 26 (66.7%) Aa | 23 (59.0%) ABb | 18 (46.2%) Bb | < 0.01 |
| *p-value (Fisher)* | 0.058 | 0.024 | < 0.01 | < 0.01 |  |

*N total per group: NE=36, EU=37, EMP=36, EB=39. Distinct uppercase letters in the same row indicate statistically significant differences within the same group over time (Friedman followed by Wilcoxon tests with Bonferroni correction, p < 0.05). Distinct lowercase letters in the same column indicate statistically significant differences between the adhesive protocols at that specific evaluation period (Fisher's Exact Test followed by pairwise comparisons, p < 0.05). * Although Fisher's Exact Test showed p < 0.05, post-hoc pairwise comparisons with Bonferroni adjustment found no significant differences among the groups.*
